# Supplementary material for: Photo-induced enhanced Raman spectroscopy for universal ultra-trace detection of explosives, pollutants and biomolecules
Source: Nat Commun. 2016 Jul 14;7:12189. doi: 10.1038/ncomms12189 (PMC4947161; doi:10.1038/ncomms12189)
Supplement: Supplementary Information — Supplementary Figures 1-11, Supplementary Table 1, Supplementary Notes 1-2 and Supplementary Methods [file ncomms12189-s1.pdf]

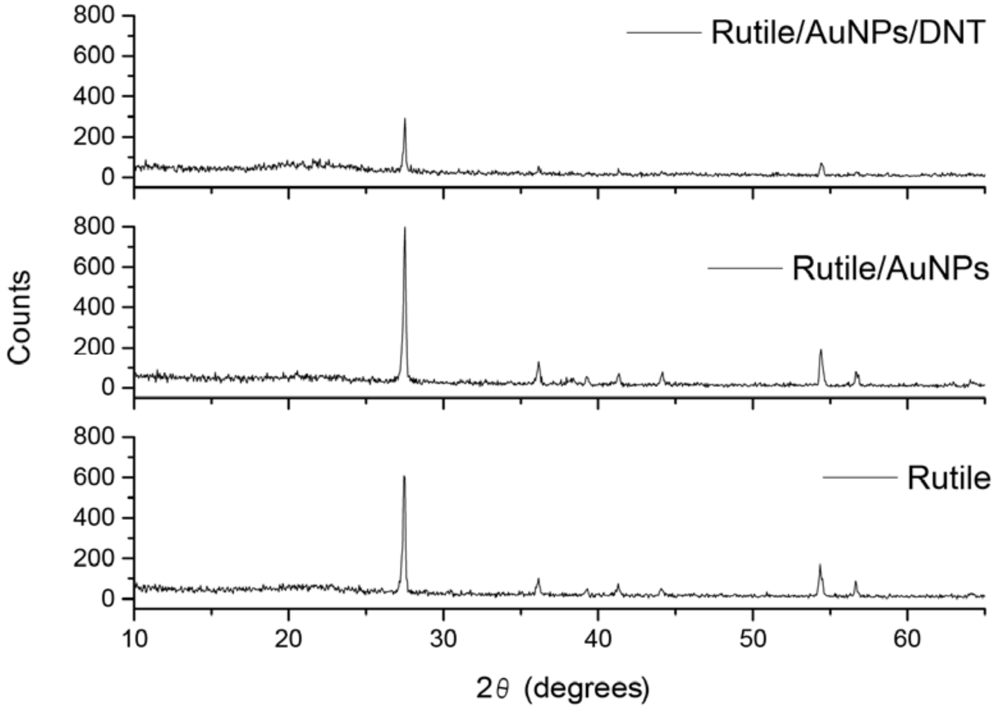

Supplementary Figure 1. Substrate X-ray diffraction patterns. Patterns show the main rutile peaks, but only very weak Au peaks from the AuNPs (gold nanoparticles). The top pattern was collected after irradiation in the presence of dinitrotoluene (DNT) as an analyte, showing no change in the peak positions, indicating no structural change in the substrate.

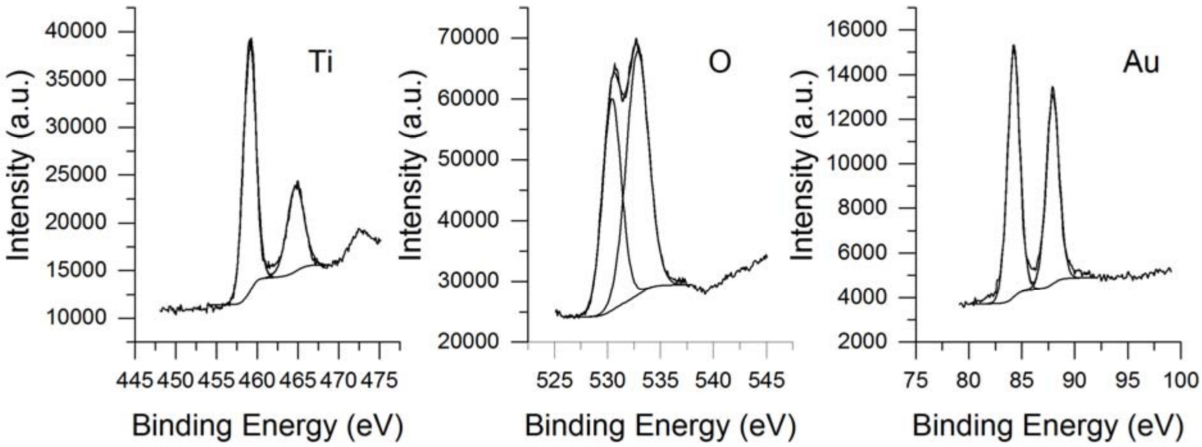

Supplementary Figure 2. Substrate XPS. XPS for Ti, O and Au on a typical PIERS substrate, showing corresponding binding energies for Ti (*2p*), O (*2p*) and Au (*2p*) environments.

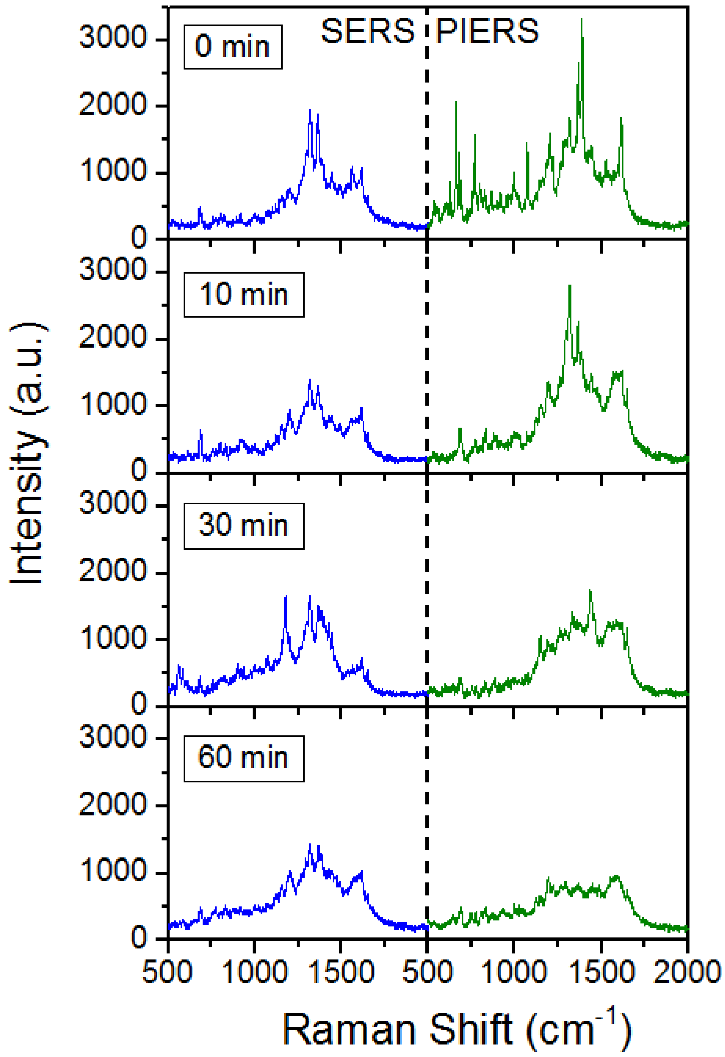

Supplementary Figure 3. Relaxation of PIERS effect. A substrate was split in two, and half was pre-irradiated with UV. DNT was deposited on both and SERS (blue) and PIERS (green) spectra were measured. The substrates were then both kept in the dark between measurements, and the average decay in spectral intensity is given in Fig. 3c in the main paper.

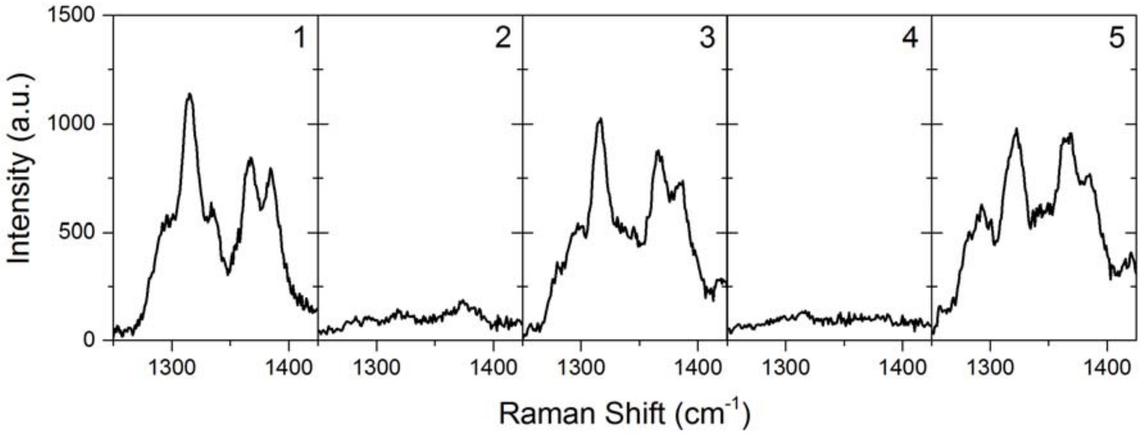

Supplementary Figure 4. Cycled PIERS spectra for DNT. PIERS spectra of DNT with 3 hr irradiation cycles between each measurement, and DNT freshly deposited before plots 1, 3 and 5.

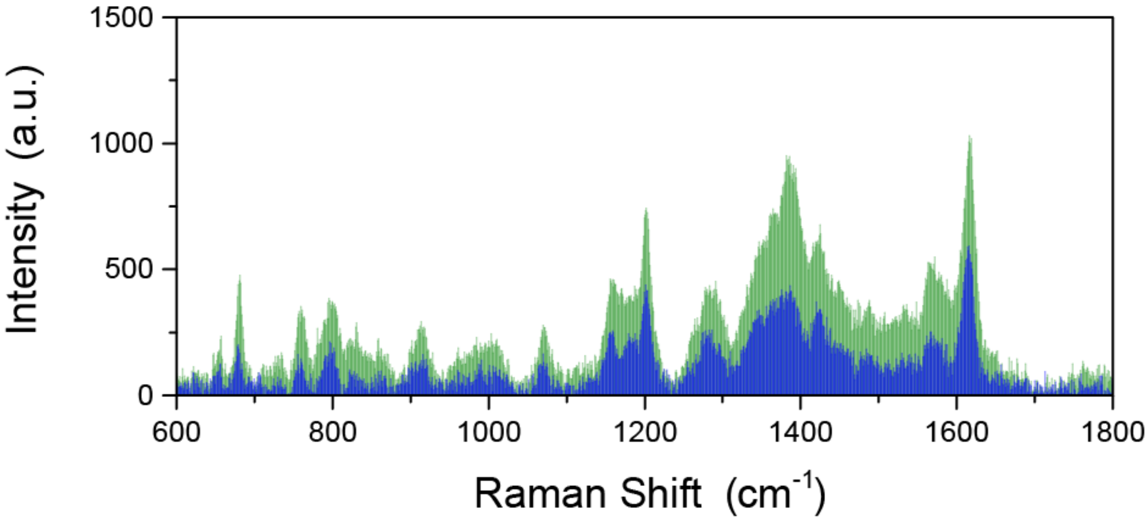

Supplementary Figure 5. PIERS of 10^{-9} M DNT on SiO_2. The SERS spectrum (blue) and the PIERS spectrum, after UV irradiation (green) are shown. An SiO_2 film treated with AuNPs was irradiated for 4 hours with 254 nm UV-light. Very little enhancement was observed as expected. However, a small increase is seen as a few high energy photons potentially transfer enough energy create a small number of excitons.

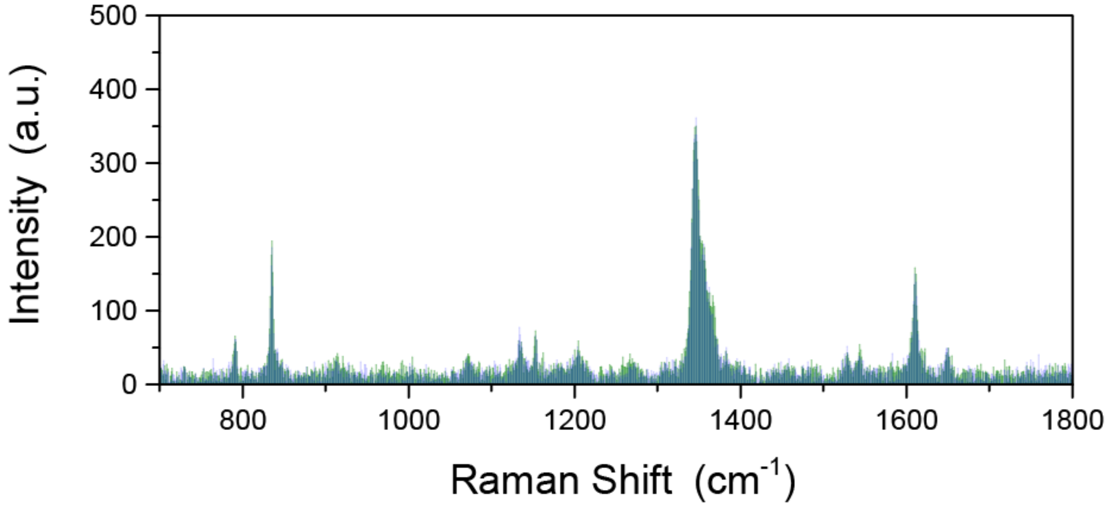

Supplementary Figure 6. PIERS without nanoparticles. Raman spectra of DNT on non-irradiated (blue) and pre-irradiated (green) TiO_2 without metallic nanoparticles on the surface. It is notable that there is low spectral intensity and no difference between conditions.

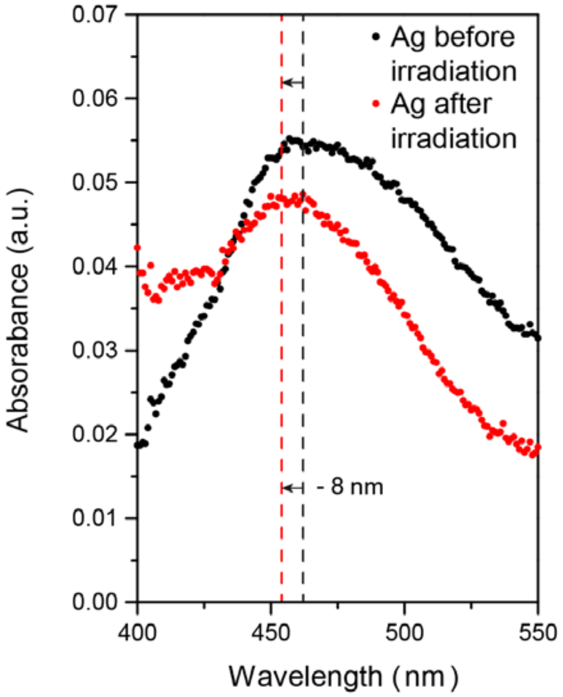

Supplementary Figure 7. Spectral shifting with Ag nanoparticles. UV-visible spectrum of AgNPs on TiO_2 showing blue shift on UV pre-irradiation. The initial LSPR of the AgNPs is shifted from their solution value (428 nm) due to the change in refractive index. This is similar to the gold case highlighted in Fig. 3b in the main text.

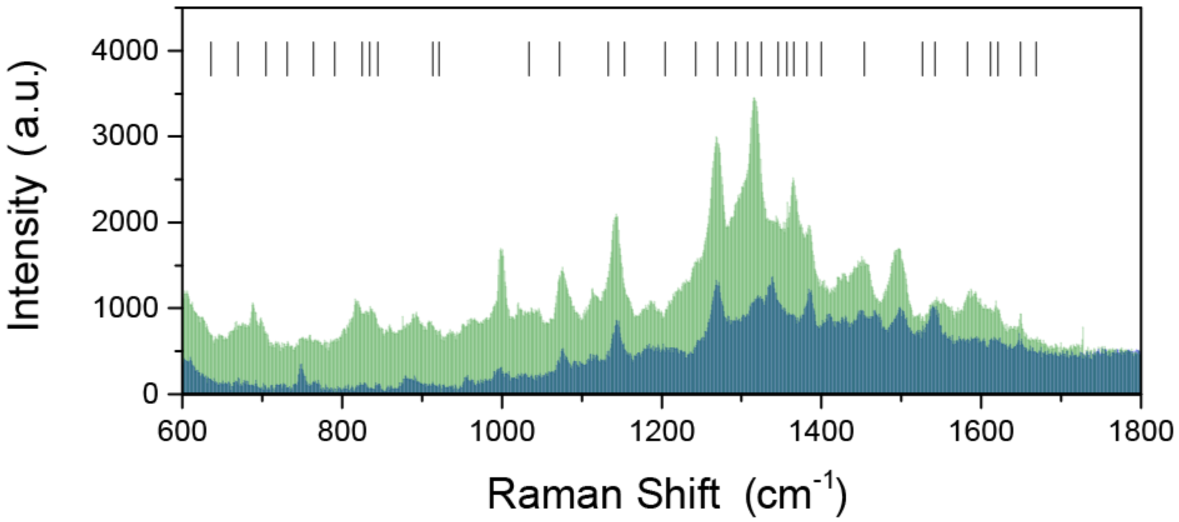

Supplementary Figure 8. PIERS with Ag nanoparticles. PIERS (green) vs SERS (blue) of 10^{-9} M DNT on TiO_2 (R) with ~ 60 nm AgNPs. Black lines are the powder spectra maxima. The enhancement effect is similar to that achieved by AuNPs as the Ag work function is also below the energy of the TiO_2 conduction band.

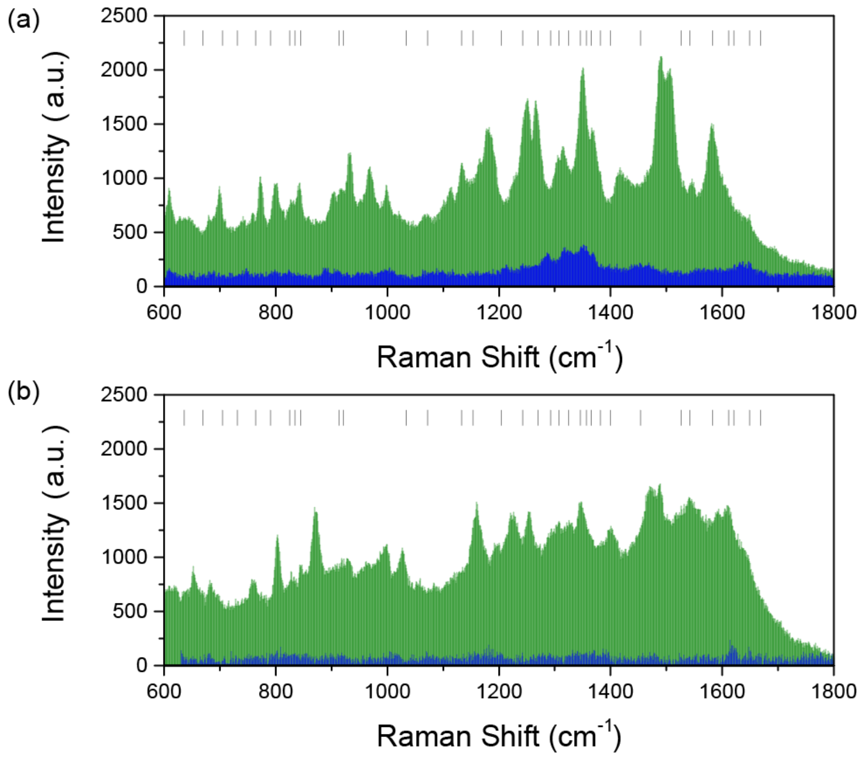

Supplementary Figure 9. Ultra-trace PIERS. PIERS spectra of DNT at ultra-trace levels (a) 10^{-12} M and (b) 10^{-15} M. Black lines are the powder spectra, blue the normal SERS spectrum and green the PIERS spectra. Similar peaks are visible, but in the latter they are more obscured by spectral noise.

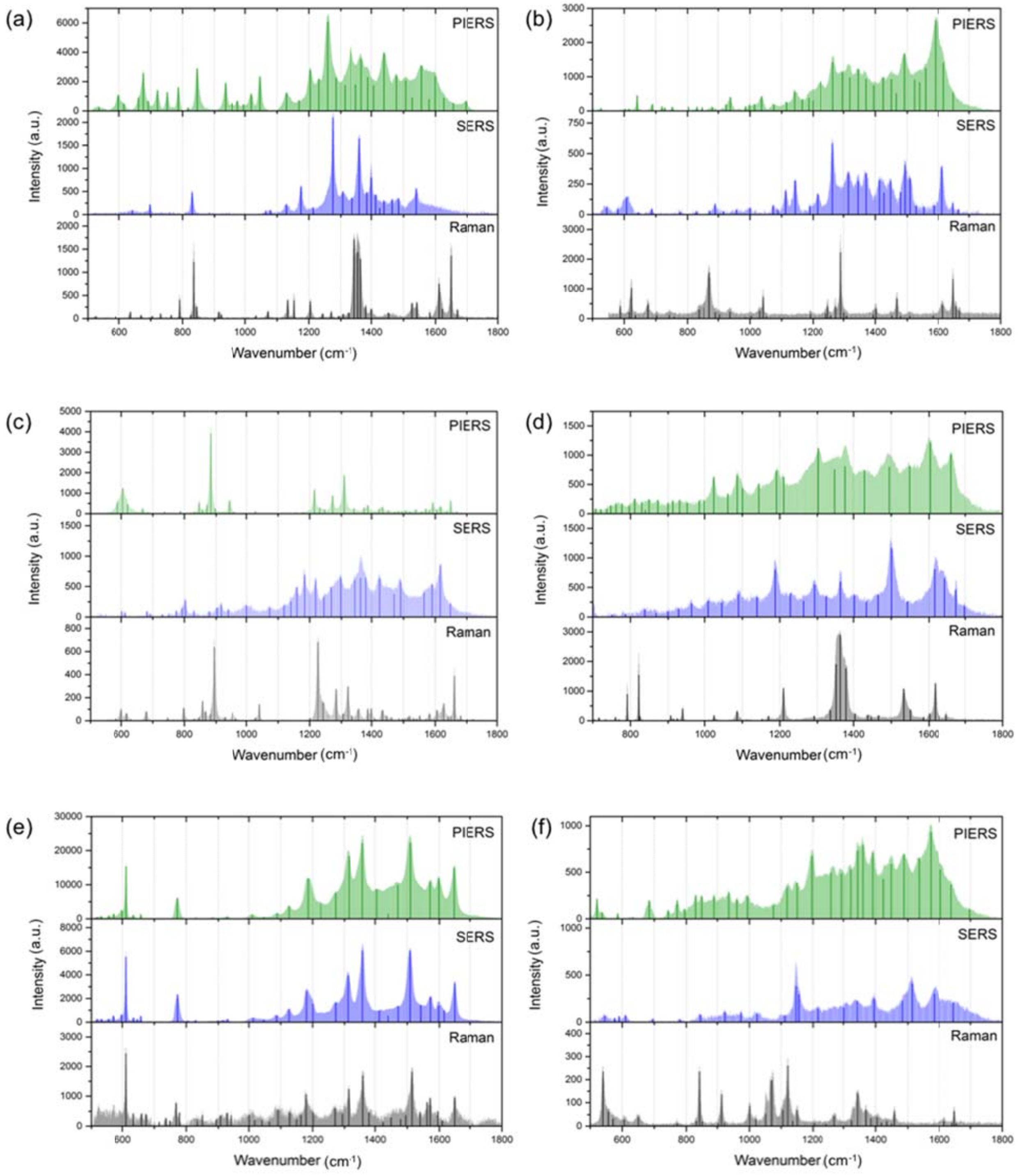

Supplementary Figure 10. Full Raman, SERS and PIERS spectra for analytes. Full Raman, SERS and PIERS spectra for all analytes described in this paper: (a) DNT 10^{-9} M, (b) PETN 10^{-5} M, (c) RDX 10^{-5} M, (d) TNT 10^{-5} M, (e) Rh6G 10^{-7} M, (f) Glucose 10^{-5} M. Broadening of the background, and a few spectral shifts suggest some decomposition of the analyte, and we acknowledge there is potential for the products of this process to give additional Raman spectra – however the spectral shapes collected were highly repeatable, and so fingerprinting by this technique is possible, just as with SERS in comparison to simple Raman spectra.

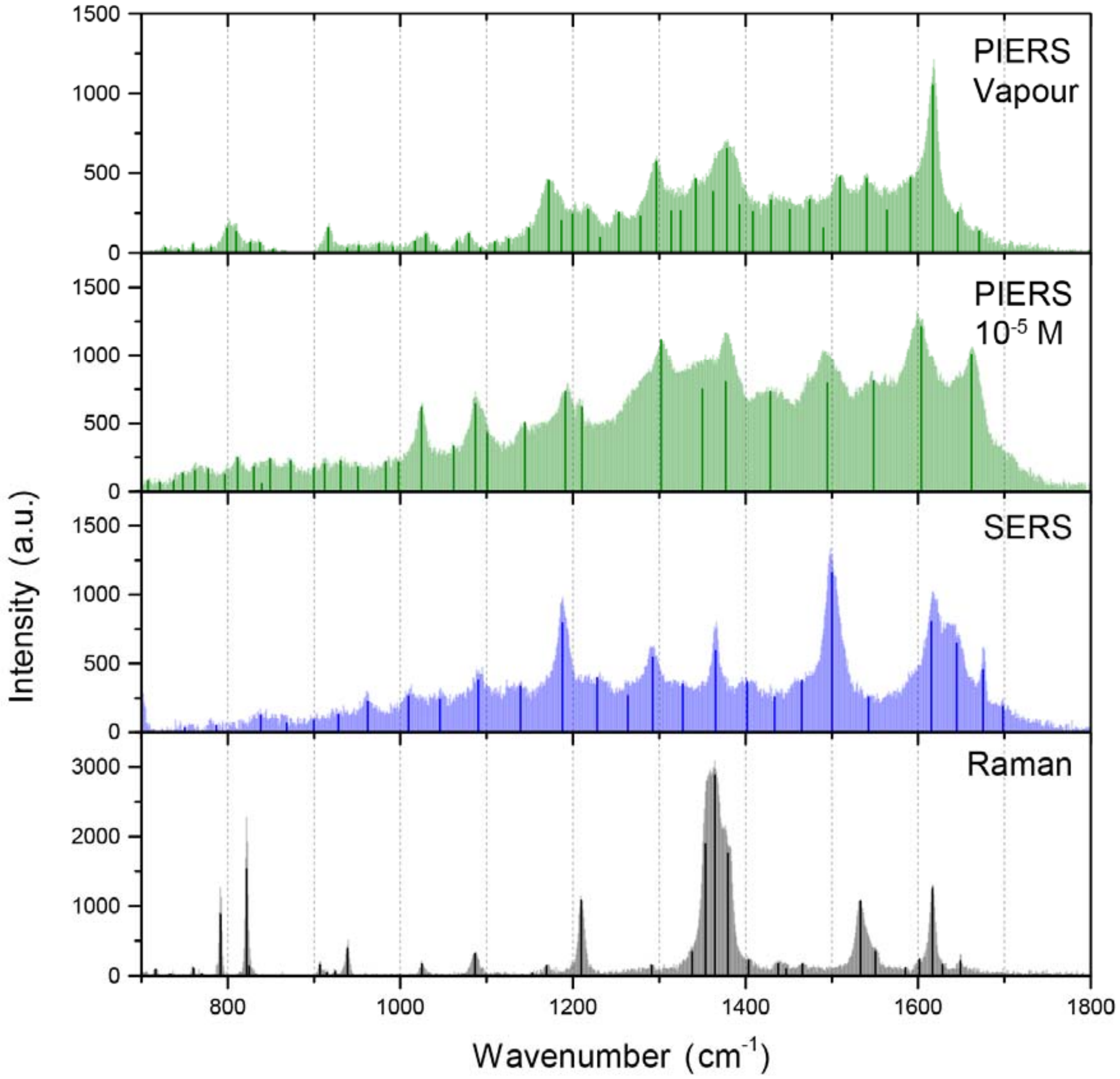

Supplementary Figure 11. Vapour detection with PIERS. Comparison of PIERS (green) spectra for solution and vapour TNT, showing similarity between the spectra and the solution SERS (blue) and powder spectrum (black). Thick lines show positions of spectral maxima. TNT vapour concentration estimated in Supplementary Note 2.

Supplementary Table 1. Key Raman resonances and their enhancement.

Analyte	$\bar{\nu}_i$ (Raman) [cm^{-1}]	Intensity (a.u.)	$\bar{\nu}_i$ (SERS) [cm^{-1}]	Intensity (a.u.)	AEF	$\bar{\nu}_i$ (PIERS) [cm^{-1}]	Intensity (a.u.)	AEF	PIERS /SERS
**R6G**	611	2602	611	5608	2.16×10^{7}	610	15543	5.97×10^{7}	2.76
	1178	625	1180	2737	4.38×10^{7}	1183	12000	1.92×10^{8}	2.94
	1361	1861	1361	5863	3.15×10^{7}	1360	24560	1.32×10^{8}	4.19
	1516	1970	1513	6151	3.12×10^{7}	1510	24470	1.24×10^{8}	3.97
**DNT**	832	550	831	750	1.36×10^{9}	843	2850	5.2×10^{9}	3.82
	1270	100	1274	1375	1.37×10^{10}	1461	4750	4.75×10^{10}	3.47
	1360	640	1362	1400	2.19×10^{9}	1364	2000	3.12×10^{9}	1.42
**TNT**	1087	145	1089	400	2.76×10^{5}	1087	1627	1.12×10^{6}	4.06
	1365	700	1365	650	9.29×10^{4}	1378	2356	3.37×10^{5}	3.63
	1614	380	1616	800	2.10×10^{5}	1605	2660	7.00×10^{5}	2.24
**RDX**	885	403	900	225	5.58×10^{4}	890	4563	1.13×10^{6}	20.25
	1215	433	1219	415	9.58×10^{4}	1216	1424	3.29×10^{5}	3.43
	1307	165	1301	390	2.36×10^{5}	1309	2177	1.32×10^{6}	5.59
**PETN**	622	270	615	190	7.04×10^{4}	644	600	2.22×10^{5}	3.15
	1289	575	1282	620	1.07×10^{5}	1281	1275	2.21×10^{5}	2.06
	1679	320	1639	480	1.50×10^{5}	1621	2890	9.03×10^{5}	6.02
**Glucose**	982	120	997	155	1.29×10^{9}	983	1290	1.08×10^{10}	8.73
	1053	250	1071	120	4.80×10^{8}	1070	4180	1.67×10^{10}	34.79
	1426	90	1425	140	1.55×10^{9}	1428	3010	1.58×10^{10}	10.20

Table of sample Raman, SERS and PIERS spectral resonances for analytes, and their enhancement factors (EFs). EFs are calculated using the equation given in Supplementary Note 1.

Supplementary Note 1. Calculation of Enhancement Factors.

EFs were calculated using the analytical enhancement factor (*AEF*) that can be defined as the following equation:

$$AEF = \frac{I_{\mathrm{SERS}} \times C_{\mathrm{RS}}}{I_{\mathrm{RS}} \times C_{\mathrm{SERS}}} \quad \text{(Supplementary Eqn. 1)}$$

Where I_{SERS} and I_{RS} are the intensities of SERS and Raman bands respectively, (I_{PIERS} uses for the intensities of PIERS bands), and C_{RS}, and C_{SERS} are the corresponding analyte concentrations in the Raman and SERS measurements, respectively, and C_{PIERS} in PIERS bands. The analyte concentration were 10^{-7} M for R6G, 10^{-9} M for DNT and glucose, 10^{-5} M for TNT, RDX, and PETN.

Supplementary Note 2. Estimation of TNT vapour concentration.

TNT concentration was calculated from the vapour pressure at 25 ^{o}C using the vapour pressure of 7.3 x 10^{-4} Pa as follows:

$$Part\ conc. = \frac{Vapour\ pressure}{Atmospheric\ pressure} \quad \text{(Supplementary Eqn. 2)}$$

This could then be converted into a rough molar concentration using the mass of TNT (227.13 g mol^{-1}) and 1 ppm = 1 mg L^{-1}.

Supplementary Methods

Titanium tetrachloride ($TiCl_4$, 99%) and ethyl acetate ($C_4H_8O_2$, 99.8%), both from Sigma-Aldrich, were used as metal and oxygen sources, respectively. All the components of the CVD apparatus were kept at high temperature (200 ºC). The precursors were heated independently in stainless steel bubblers and carried under controlled flows using pre-heated nitrogen gas (supplied by BOC). The precursors were mixed in a stainless steel chamber (250 ºC) before accessing the CVD reactor and then plain nitrogen flow dragged the gas precursors' mixture through a triple baffle manifold to generate a wide laminar flow. The cold-wall CVD reactor consists of a 320 mm-long heating graphite block accommodated in a quartz tube, with three inserted Whatman heater cartridges. The temperature of the entire system was controlled by Pt-Rh thermocouples.

In a typical deposition, bubbler temperatures and gas flows of the precursors were set to 1.2 L min^{-1}/70 °C and 0.25 L min^{-1}/40 °C for $TiCl_4$ and $C_4H_8O_2$, respectively. The TiO_2 film was deposited (growth rate, 0.45 – 0.5 μm min^{-1}) on quartz slides (25x25 mm, Multi-Lab) at 500 °C and then annealed to 900 °C for 10 h. Due to temperature limitation of the CVD (chemical vapour deposition) rig, pure rutile films were obtained after heat treatment (1050 °C, 10 h) of an anatase film deposited on quartz slides. X-ray diffraction and Raman spectroscopy confirmed the presence of pure rutile; no traces of anatase were detected.

Comparative SiO_2 coated glass was obtained from Pilkington NSG in the form of commercial barrier glass.

AuNPs were produced using the standard Turkevich-Frens procedure to give an average size of 26.6 nm, with standard deviation (s.d.) of 5 nm. $HAuCl_4$ (120 mg) was dissolved in 250 mL of boiling water. A 1% (w/w) sodium citrate solution (25 mL) was added and the reaction kept boiling for 1 hour. AuNPs were precipitated by ultra-centrifugation at 12000 x g, and re-suspended in MeOH at an approximate concentration of 4.2 x 10^{-10} M (by UV-vis). The solution was drop cast onto the substrates, followed by air drying, to give a rough coverage of 250 AuNPs μm^{-2}.

AgNPs were produced by the standard Turkevich method with a single modification in the pH values along the reaction. Initially, NaOH (0.1 M) was used to adjust the pH to 7.7 of an aqueous solution of tri-sodium citrate solution (7 mM). This solution was heated until it started boiling and then 1 mL of 0.1 M aqueous silver nitrate was added. After stirring the reaction solution for 5 minutes the pH was adjusted to 6.1 by addition of HNO_3 in order to slow down the reaction and get better shape and size distribution. The reaction was complete after 30 minutes, giving rise to particles of 58nm with s.d. of 14 nm.

UV-visible (UV-Vis) spectra were collected on Perkin Elmer Lambda 25 and 950 systems in absorbance or reflectance mode, and a Shimadzu UV-2550 instrument. XRD patterns were collected between 10° and 65° with a Bruker-Axs D8 System, with Cu Kα source (1.54 Å). The incident beam angle was 1°. X-ray Photoelectron Spectroscopy (XPS) measurements were performed with a Thermo monochromated aluminium k-alfa photoelectron spectrometer, using monochromic Al-Kα radiation

(1486.7 eV). Survey scans were collected in the range of 0 - 1200 eV. High-resolution peaks were used for the principal peaks of Ti (2p), O (1s), C (1s) and Au (4f). The peaks were modelled using sensitivity factors to calculate the film composition. The area underneath these bands is an indication of the concentration of element within the region of analysis (spot size 400 µm). Data was analysed with CasaXPS software. Nanoparticles and films were imaged on a Jeol 6700F FEG SEM operating at 5 kV and a Jeol 2100 TEM operating at 200 kV with a Gatan Orius digital camera. Particle sizing was performed using ImageJ software.
